# Detecting recombination in evolving nucleotide sequences

**DOI:** 10.1186/1471-2105-7-412

**Published:** 2006-09-18

**Authors:** Cheong Xin Chan, Robert G Beiko, Mark A Ragan

**Affiliations:** 1 ARC Centre in Bioinformatics and Institute for Molecular Bioscience, the University of Queensland, Brisbane, QLD 4072, Australia

## Abstract

**Background:**

Genetic recombination can produce heterogeneous phylogenetic histories within a set of homologous genes. These recombination events can be obscured by subsequent residue substitutions, which consequently complicate their detection. While there are many algorithms for the identification of recombination events, little is known about the effects of subsequent substitutions on the accuracy of available recombination-detection approaches.

**Results:**

We assessed the effect of subsequent substitutions on the detection of simulated recombination events within sets of four nucleotide sequences under a homogeneous evolutionary model. The amount of subsequent substitutions per site, prior evolutionary history of the sequences, and reciprocality or non-reciprocality of the recombination event all affected the accuracy of the recombination-detecting programs examined. Bayesian phylogenetic-based approaches showed high accuracy in detecting evidence of recombination event and in identifying recombination breakpoints. These approaches were less sensitive to parameter settings than other methods we tested, making them easier to apply to various data sets in a consistent manner.

**Conclusion:**

Post-recombination substitutions tend to diminish the predictive accuracy of recombination-detecting programs. The best method for detecting recombined regions is not necessarily the most accurate in identifying recombination breakpoints. For difficult detection problems involving highly divergent sequences or large data sets, different types of approach can be run in succession to increase efficiency, and can potentially yield better predictive accuracy than any single method used in isolation.

## Background

A homologous recombination event between two DNA sequences can be either reciprocal or non-reciprocal. In reciprocal recombination, genetic information is transferred or exchanged between two similar DNA sequences. In non-reciprocal recombination, a contiguous region of DNA is replaced by, rather than exchanged with, the transferred region. Both types of recombination are a consequence of the DNA mismatch repair mechanism which protects genetic information from damage. Gene conversion, for example, is a cross-over process between homologous sequences in which a DNA strand replaces a damaged partner DNA strand with a copy of its own sequence [1]. A number of models describe the mechanisms of recombination, addressing issues of strand breakage, displacement and extension, and mismatch repair in double-stranded DNA [2-4]. Gene conversion events can lead to reshuffling of parental open reading frames, or of structural and functional motifs within protein domains, and these can generate a gene with novel functions [5,6]. Therefore, reciprocal and non-reciprocal recombination events are important mechanisms in the creation of genetic diversity [7].

Recombination events have been inferred in prokaryotes [8-10], unicellular eukaryotes [11,12] and multicellular eukaryotes [13,14]. Homologous recombination has contributed to the evolution and functional divergence of multi-gene families such as the β-globin gene family [15], heat shock proteins [16,17] and the major histocompatibility complex gene family [18,19]. While most cases reflect recombination between DNA sequences within a genome, recombining DNA can also come from the external environment of a cell, which results in the acquisition of foreign DNA by a genome [20,21]. If lateral genetic transfer via homologous recombination is a significant contributor to prokaryotic evolution [22,23], the detection of recombination events will be essential in the inference of phylogenetic relationships among genomes [24].

Elucidating patterns of genetic transfer will enhance our understanding of the role selective forces play in shaping genomes. Homologous recombination events can produce genes with mosaic evolutionary histories in which the underlying evolutionary pattern is not a tree but a network [25,26]; such an evolutionary pattern confounds analyses that assume a common evolutionary path for every component of a biological sequence. The task of delineating recombination events is hard for two major reasons. Firstly, if the recombining sequences are too similar, subsequent detection of the event may be impossible due to the lack of 'signal' to distinguish among sequences. Secondly, evolutionary events that occur after recombination will tend to obscure the true relationships between sequences. Homologous recombination events can overwrite previous such events, fragmenting the regions with consistent evolutionary histories until the events cannot be distinguished with confidence [27]. Sequence substitutions after a recombination event will diminish the apparent similarity between fragments of a gene and their closest relatives in other sequences; this phenomenon has been shown to influence the accuracy of phylogenetic inference [28].

A number of approaches are available to detect evidence of recombination events and/or to identify the recombination breakpoints. They are classified into different categories based on the algorithms used [29]. *Distance-based *methods generate statistics of genetic distances with the use of a sliding window along a set of aligned sequences [30,31]. Abnormal inversions of distance patterns are detected without reference to the underlying phylogenetic relationship among the sequences. *Phylogenetic-based *methods are based on the detection of alignment partitions with discordant phylogenetic relationships [32]. In *compatibility-based *methods, inference of recombination and phylogenetic incongruence is based on parsimoniously informative sites identified within an alignment [33-35]. *Substitution distribution-based *methods detect regions within a set of sequences that are significantly similar or clustered together, with the level of significance based on a modelled statistical distribution of nucleotide substitution [36]. Computer simulations and empirical data have been used to evaluate the performance of a number of available methods in detecting and analysing recombination events based on the amount of recombination and sequence divergence [29,37,38]. A uniform outcome from these studies is that compatibility-based and substitution-based methods perform better than the phylogenetic-based approaches. Furthermore, Posada and Crandall [28] demonstrated that phylogeny reconstruction from sequences could be biased owing to the reciprocality and the age of a recombination event, as well as the parental divergence of the sequences involved in the event. These studies suggested that conclusions about recombination should not be drawn on the basis of a single method due to biases of different approaches to the nature of the dataset, e.g. some methods were found to detect far fewer recombination events and breakpoints than expected [38,39]. Using simulated sequence data, we examined the effect of subsequent substitution after a recombination event on the prediction accuracy of different recombination-detecting programs, within the simplified framework of homogeneous substitution rate and nucleotide composition throughout the lineages.

## Results

**Figure 1:**
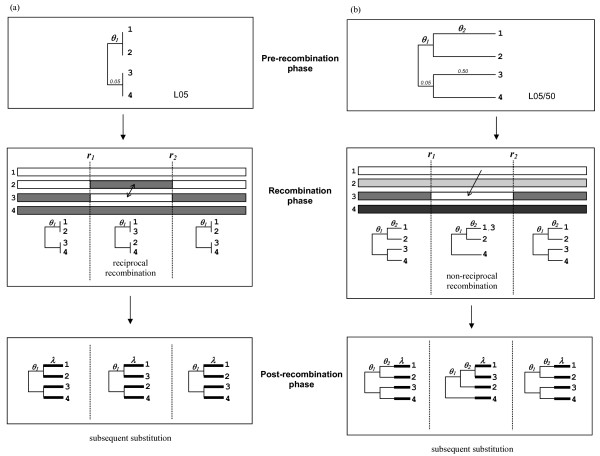
**Simulation of four-taxon sequence evolution with a single (a) reciprocal or (b) non-reciprocal recombination event**. The simulation of sequence substitution was divided into three phases: a pre-recombination phase representing the evolutionary history prior to recombination; a recombination phase in which the recombination event occurs; and a post-recombination phase representing subsequent evolution after the recombination event. The recombination event was either (a) reciprocal or (b) non-reciprocal in nature: in both cases recombination was performed between a predefined pair of breakpoints *r_1 _*and *r_2_*. The lineages of length *θ_1 _*and *θ_2 _*represent the pre- and post-speciation lineage respectively, at the pre-recombination phase. In a reciprocal recombination event, the segments of sequences 2 and 3 between the recombination breakpoints were exchanged. As a consequence, the canonical relationships between sequences were preserved in the non-recombined region, with sequence pairs (1,2) and (3,4) most-similar to one another, while in the recombined region sequence pairs (1,3) and (2,4) were most-similar. In a non-reciprocal recombination event, the region of sequence 3 between breakpoints *r_1 _*and *r_2 _*was replaced by the homologous region from sequence 1, while sequences 2 and 4 did not donate or receive any genetic material. The recombined regions of sequences 1 and 3 were identical immediately after a non-reciprocal event. During the post-recombination phase, subsequent substitutions were simulated no each sequence independently at *λ *substitutions per site.

We simulated the evolution of four-sequence sets with 1000 nucleotides (nt) per sequence under a homogeneous evolutionary model as illustrated in Figure 1. Each simulation consisted of three phases. During the pre-recombination phase, sequences were simulated along lineages of length *θ*. A lineage length is defined by the average number of substitutions per site. The lineages of length *θ_1 _*and *θ_2 _*in the non-reciprocal set represent the pre- and post-speciation lineage, respectively. The notation 'L05/50' refers to a set of four sequences that were simulated along a tree topology with *θ_1 _*= 0.05 and *θ_2 _*= 0.50, prior to the simulated recombination event. At the recombination phase, an exchange of the recombined region (between breakpoints *r_1 _*and *r_2_*) was performed between sequences 2 and 3 to simulate a reciprocal event (Figure 1a). To simulate non-reciprocal recombination, the region in sequence 3 between *r_1 _*and *r_2 _*was replaced with the corresponding region from sequence 1 (Figure 1b). The recombined regions of sequences 1 and 3 were identical immediately after a non-reciprocal event, leading to a change of tree topology in which 1 and 3 were sister taxa, and taxon 4 was separated from the root by a single branch of length *θ_1 _*+ *θ_2_*. During the post-recombination phase, subsequent nucleotide substitutions were simulated independently for each sequence with *λ *substitutions per site in each lineage. For each simulation set, the same evolutionary model with equal rate of substitutions was applied to all four lineages following speciation. Within the context of reciprocal recombination, the exact point at which a recombination event occurs along the lineages after speciation cannot be distinguished. Therefore, reciprocal recombination was performed immediately after the speciation event at the end of lineage *θ_1_*. While *θ_2 _*is the length of the branches immediately preceding a non-reciprocal recombination event, *θ_1 _*is the lineage length immediately preceding a reciprocal event. Lambda (*λ*) represents the amount of independent subsequent substitutions after a recombination event in both reciprocal and non-reciprocal sets.

The five programs compared in this study were GENECONV (substitution distribution-based) [36], RecPars (phylogenetic-based) [40], Reticulate (compatibility-based) [33], BARCE [41] and DualBrothers [42] (both Bayesian phylogenetic-based). These programs were selected based on their capabilities for large-scale automated analyses, their usage within the scientific community and/or strong performance within their class of algorithm in recent reviews [29,37,38]. A brief overview of our methods for calculating prediction accuracy is provided below, with further details in the Methods section. The phylogeny of the recombined region between points *r_1 _*and *r_2 _*in each sequence set was assessed by Bayesian inference using a Markov chain Monte Carlo (MCMC) approach, implemented in MRBAYES [43]. As the amount of subsequent substitutions increases, the phylogenetic signal becomes weaker, and the Bayesian posterior probability (BPP) of the tree topology is expected to decrease. Failure to assign high BPP to the correct topology within the recombined region indicates a loss of phylogenetic signal, and suggests that statistical searches for recombination events or breakpoints, particularly those based on phylogenetic relationships, may be futile.

### Substitution distribution- and phylogenetic-based approaches

**Figure 2:**
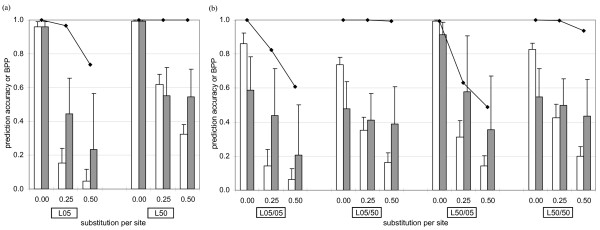
**Prediction accuracy of RecPars and GENECONV on the (a) reciprocal set and (b) non-reciprocal set**. On the Y-axis of each graph, hollow bars represent the prediction accuracy of GENECONV, solid bars represent the prediction accuracy of RecPars, while the lines with filled diamonds () indicate BPP of the tree topology within the recombined region, inferred by MRBAYES. The X-axis on each graph represents substitutions per site simulated after recombination (*λ*) at different test case of prior evolutionary history. The error bars represent standard deviations of the data collected. See text for details.

The prediction accuracy of GENECONV and RecPars was determined based on separate calculations of the number of correctly assigned residues within the recombined and the non-recombined regions (see Methods section for details). Perfect classification of both the recombined and non-recombined regions would yield a score of 1.0, while the assignment of a single tree topology to the entire alignment (no recombination events inferred) would lead to a score of 0. The prediction accuracy of the two programs and BPP assigned to the correct tree topology for the recombined region are depicted in Figure 2 for simulations of (a) reciprocal and (b) non-reciprocal events and different amounts of sequence substitution. When *λ *was ≥ 0.25 substitutions per site, RecPars in general showed higher accuracy (e.g. 0.4458 at *λ *= 0.25 in L05) than GENECONV (e.g. 0.1532 at *λ *= 0.25 in L05). GENECONV, with lower standard deviations (maximum standard deviation of 0.09 compared to the equivalent of 0.32 in RecPars), was more consistent across simulated replicates. The accuracy of both programs showed an inverse relationship with the increase of subsequent substitution after recombination. The observation can be related to the fact that GENECONV and RecPars identified multiple fragments rather than a whole fragment within the recombined region when *λ *was ≥ 0.25 substitutions per site.

The phylogenetic signal of the recombined region was stronger when the recombining sequences were more divergent, i.e. when the immediate preceding lineage length leading up to the recombination event was longer. The BPP values obtained with MRBAYES were > 0.90 in all cases when the immediate preceding lineage was at 0.50 substitutions per site, even when subsequent substitutions were high (BPP in L05/50: 0.99, L50/50: 0.94 and L50: 1.00 at *λ *= 0.50). The recombination signal and prediction accuracy decreased more rapidly in response to increasing *λ *when the recombining sequences were more similar to one another (L05/05, L50/05 and L05).

### Compatibility-based approach

**Figure 3:**
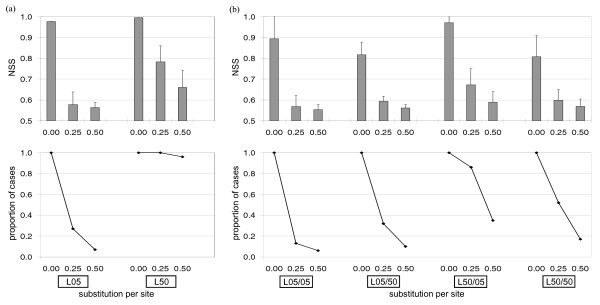
**Performance accuracy of Reticulate on the (a) reciprocal set and (b) non-reciprocal set**. The X-axis of each graph represents substitution per site after recombination (*λ*) at different test cases of prior evolutionary history. The bars represent the neighbour-similarity scores (NSS) with error bars showing standard deviation of the scores. The line () represents the proportion of simulation sets in which clustering is significant (*p*-value ≤ 0.05).

Reticulate generates a compatibility matrix displaying the most-parsimonious relationships among sequences for each informative site in a sequence alignment. Two informative sites are considered compatible if they can be explained by the same phylogenetic tree with the most parsimonious change [33]. A cluster of mutually compatible sites that is incompatible with sites outside the cluster suggests a recombination event. The neighbour similarity score (NSS) has a range between 0.5 and 1.0, and represents the extent to which mutually compatible sites are found in contiguous blocks. This score was used as the criterion of prediction accuracy. The relationship between site clustering efficiency in Reticulate and the extent of subsequent substitution occurring after recombination is shown in Figure 3. In all cases, the NSS approached the minimal value of 0.5 as subsequent substitution (*λ*) reached 0.5 substitutions per site.

Within the reciprocal set (Figure 3a), when recombining sequences were more dissimilar (L50), Reticulate showed higher NSS (0.9950 at *λ *= 0.00) and higher proportions of sets with significant clustering (1.00 at *λ *= 0.25) compared to L05 (NSS 0.9760 at *λ *= 0.00; proportion 0.27 at *λ *= 0.25). In comparison to the reciprocal set, lower NSS were obtained within the non-reciprocal set even when no subsequent substitution was simulated e.g. NSS in L05/50: 0.8174 and L50/50: 0.8078 at *λ *= 0.00. When *λ *reached 0.50 and the immediate preceding lineage length (*θ_2_*) was long, the proportion of significant clustering within the non-reciprocal set was low (e.g. 0.10 in L05/50 and 0.17 in L50/50). This can be explained by the resulting identical sequence fragments in a non-reciprocal event, which yielded fewer parsimoniously informative sites in the alignment. The measure of statistical significance within a set of data is lower when the sample size is smaller, because the probability of obtaining a result by chance is higher. Therefore, as fewer parsimoniously informative sites were being considered in a non-reciprocal event, the clustering efficiency of sites in the compatibility matrix was lower.

### Bayesian phylogenetic-based approach

**Figure 4:**
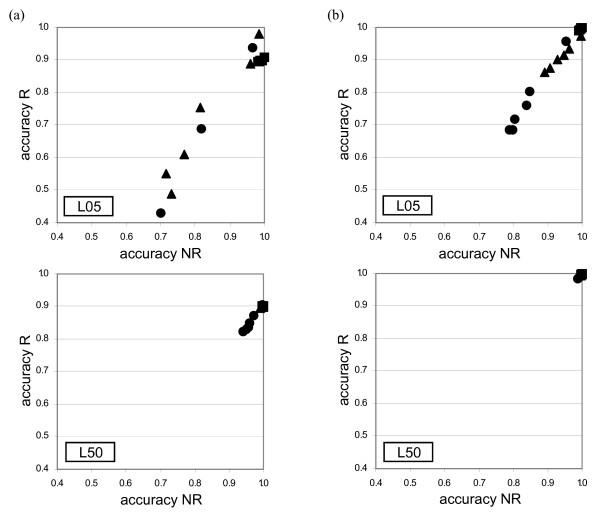
**Prediction accuracy of (a) BARCE and (b) DualBrothers on the reciprocal set**. The Y-axis of each graph represents prediction accuracy within the recombined region, while the X-axis represents prediction accuracy within the non-recombined region. Each data series on a graph represents *λ*, the average number of substitution per site simulated after recombination [■:0.00; ▲: 0.25; ●: 0.50], and each data point represents accuracy obtained at a particular probability threshold level. See text for details.

For each site (column) of an alignment, BARCE and DualBrothers assign BPP to possible tree topologies. Average accuracies within the recombined and non-recombined regions were computed in a manner similar to RecPars and GENECONV above, but each site prediction contributed to the accuracy only if one tree topology had a BPP greater than a specified threshold, as shown in Figures 4 (for the reciprocal set) and 5 (for the non-reciprocal set). Experimental sets showing high accuracy in both recombined and non-recombined regions (in both axes X and Y on the graphs) are an indication of desirable performance. In general, the prediction accuracy of BARCE and DualBrothers are better in comparison with the other methods examined. Both programs tended to identify a single contiguous recombined region in comparison to the multiple fragments identified by GENECONV and RecPars.

**Figure 5:**
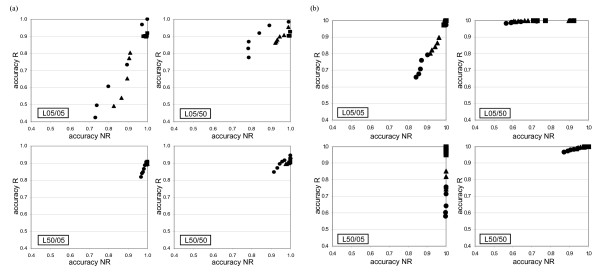
**Prediction accuracy of (a) BARCE and (b) DualBrothers on the non-reciprocal set**. The Y-axis of each graph represents prediction accuracy within the recombined region, while the X-axis represents prediction accuracy within the non-recombined region. Each data series on a graph represents substitution per site after recombination (*λ*) [■:0.00; ▲: 0.25; ●: 0.50], and each data point represents accuracy obtained at a particular probability threshold level. See text for details.

For the reciprocal sets (Figure 4), both BARCE and DualBrothers showed high accuracy when recombining sequences were more divergent (L50), with DualBrothers showing higher accuracy than BARCE; within the recombined region, DualBrothers showed a minimum accuracy of 0.98 and BARCE showed a minimum accuracy of 0.82 when *λ *= 0.50.

A slightly different trend was observed in the non-reciprocal set (Figure 5). Consistent with the observation in Figure 1, when the immediate preceding lineage length (*θ_2_*) was short (L05/05 and L50/05), the phylogenetic signal of the recombined region was diminished to a greater extent by the resulting identical sequence fragments. BARCE and DualBrothers showed lower accuracy in recovering this region than the non-recombined region, especially when *λ *was high. For instance in L50/05, DualBrothers showed lower accuracy in recovering the recombined region (minimum accuracy 0.58; *λ *= 0.50) than in recovering the non-recombined region (minimum accuracy 0.99; *λ *= 0.50). The exact opposite trend was observed in L05/50 when recombining sequences were more divergent, with accuracy in recovering the recombined region higher (minimum accuracy 0.98; *λ *= 0.50) than in recovering the non-recombined region (minimum accuracy 0.56; *λ *= 0.50). Similar bias in recovering recombined or non-recombined region was observed with BARCE, although to a lesser extent. When both primary and secondary lineage lengths of a tree were long (L50/50) prior to recombination, both programs showed high accuracy in recovering both regions, DualBrothers (e.g. minimum accuracy 0.97 within recombined region; *λ *= 0.50) more so than BARCE (e.g. minimum accuracy 0.84 within recombined region; *λ *= 0.50).

**Figure 6:**
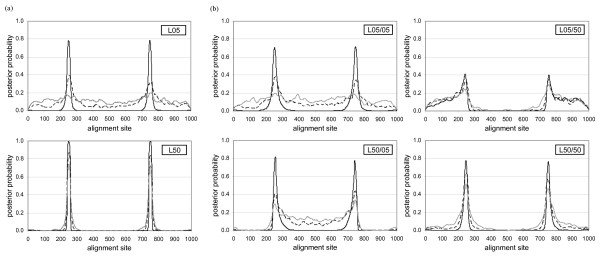
**Identification of (a) reciprocal and (b) non-reciprocal recombination breakpoints in DualBrothers**. The Y-axis of each graph represents the posterior probability of a site being proposed as a recombination breakpoint, while the X-axis represents each site on the alignment. In all cases, the simulated recombination breakpoints are at positions 250/251 and 750/751. The different lines on each graph represent the amount of subsequent substitution (*λ*) simulated (in substitutions per site): black solid line, 0.00; black dashed line, 0.25; and grey solid line, 0.50.

BARCE and DualBrothers, which use a Bayesian approach to represent the sequential relationship and interaction among different sites of the alignment, proved to be more accurate in defining recombination breakpoints as compared to the other approaches. Figure 6 shows the posterior probability of a site being proposed as a change-point of tree topology by DualBrothers across all sites in the alignment. Two sharp peaks proximate to the designated breakpoints *r_1 _*(250/251) and *r_2 _*(750/751) were obvious in all cases when no subsequent substitution was simulated after recombination. The posterior probability decreased with increasing amount of subsequent substitution. For the non-reciprocal set, the observation was consistent with Figure 5: more false positives were observed within the recombined region in L50/05, and more false positives were observed within the non-recombined region in L05/50.

The Markov chains generated by DualBrothers were always initiated from a random point in the space of trees, breakpoints and models. To assess the sensitivity of sampled breakpoint posterior probabilities to the choice of start point, a subset of our simulated datasets was used, in which DualBrothers was implemented on each simulated set ten times, each time starting at a random point (see Methods section). For the majority of parameter combinations, the posterior probability of breakpoints at each of the 1000 sites in the sequence was extremely stable (BPP range < 0.01) across all ten replicates, with higher variability observed at the 'shoulder' regions of breakpoints (BBP range < 0.10). Even higher variability was observed in parameter combinations that yielded the most difficult-to-detect breakpoints (BPP range reached 0.8 in the worst case, L05/05; *λ *= 0.50), showing that the dataset requires a much longer run to achieve stable BPPs. Nevertheless, when a given site was assigned a breakpoint BPP ≥ 0.5 in a replicate, the BPP assigned to the same site in each of the other replicates was always greater than each respective median BPP. Consequently, breakpoint identification was at worst still consistent across replicates, in spite of the high BPP variation seen for this combination of parameters.

### Sensitivity to parameter settings

**Figure 7:**
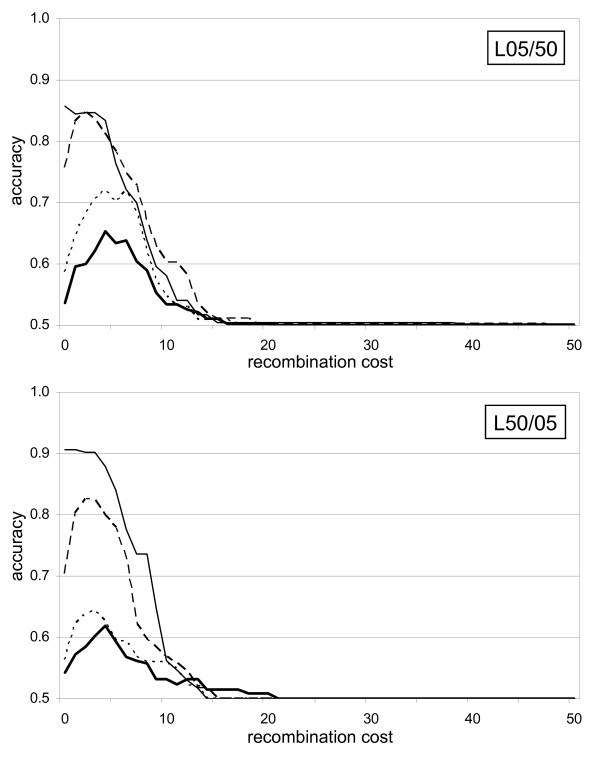
**Relationship between performance accuracy of RecPars and the recombination cost used**. The Y-axis of each graph represents the average RecPars prediction accuracy within the recombined region and within the non-recombined region. The X-axis of each graph represents the value of recombination cost used. Two prior evolutionary histories for the non-reciprocal set are shown: one where *θ_2 _*is much longer than *θ_1 _*(L05/50), and one where *θ_2 _*is much shorter than *θ_1 _*(L50/05). The different lines on each graph represent the amount of subsequent substitution (*λ*) simulated (in substitutions per site): thin solid line, 0.00; dashed line, 0.25; dotted line, 0.50; and thick solid line, 1.00.

Although RecPars tended to show higher prediction accuracy than GENECONV (Figure 2), the optimal recombination cost was determined separately for every simulated data set in this study to yield the best possible prediction accuracy. We found little consistency in the choice of optimal RecPars recombination cost across multiple simulations. When the cost of assigning a recombination breakpoint was too low for a given set of sequences, RecPars defined a great number of incongruent tree topologies across the sequences, with some regions having a length of one column within the alignment. In contrast, no recombined region was detected if the recombination cost was too high. Figure 7 shows the relationship between prediction accuracy of RecPars and the recombination cost for L05/50 and L50/05. The appearance of sharp peaks in the graphs shows that prediction accuracy is highly sensitive to the recombination cost. A similar situation applies to GENECONV with respect to setting the value of the *gscale *parameter. *Gscale *is a scaling factor of mismatch penalties on the polymorphic sites; setting *gscale *to zero prohibits fragments with internal mismatches, while *gscale *= 1 allows internal mismatches in the pairwise comparisons. Posada [37] reported that GENECONV at *gscale *setting = 0 gave misleading results in a number of divergent empirical datasets. In separate optimisation tests on the simulation sets in this study, a *gscale *value of 1 was found to recover the largest fraction of the recombined regions in the simulated datasets (results not shown).

### Relationship between simulation parameters and prediction accuracy

In this study, for each reciprocal and non-reciprocal set, the variation in prediction accuracy of recombination-detecting programs was related to a few major factors: (i) evolutionary history prior to recombination (*θ*), (ii) substitutions after the recombination event (*λ*), and (iii) parameter settings for certain programs i.e. GENECONV and RecPars. To examine the relationship between these factors and prediction accuracy, multiple linear regression (MLR) analysis was carried out for each program individually. Across all five detection programs in this study, all of the factors considered had significant effects on the prediction accuracy (*p*-values < 2.20 × 10^-16^). Since extremely low *p*-values could not be differentiated, comparison of the *t*-statistics associated with different factors was assessed to determine the relative strength of their effects on prediction accuracy. Of the factors examined, the extent of subsequent substitution had the strongest effect on prediction accuracy in all approaches, e.g. GENECONV with *t*-values -135.40 in the reciprocal set and -177.83 in the non-reciprocal set, consistent with the observed trends in Figures 2 through 6.

The overall *F *values obtained in the MLR analysis for each factor examined are associated with very small *p*-values (< 2.20 × 10^-16^), supporting the graphical evidence in Figures 2 to 7 that the effects of simulation parameters on prediction accuracy are statistically significant. The adjusted *R*^2 ^value shows the percentage of the outcome that could be explained by the factors examined in the study. The highest *R*^2 ^value (0.85; *F *value 6863; 3596 degrees of freedom in reciprocal set) was assigned to the substitution distribution-based approach (GENECONV), implying that approximately 85% of the outcome could be explained by the factors examined. The compatibility-based approach, Reticulate, showed a similarly high *R*^2 ^value (0.83). The phylogenetic-based approaches RecPars (0.58), BARCE (0.68) and DualBrothers (0.28), had lower associated *R*^2 ^values (values shown for the reciprocal set). Most of the MLR assumptions [44] were not violated by the data analysed, except for a slightly non-linear relationship observed between independent and dependent variables, and evidence for heteroscedasticity in some analyses (results not shown). Although the assumption of homoscedasticity was sometimes violated, MLR is robust to violations of this assumption when sample sizes are large, so the observed heteroscedasticity is unlikely to have a strong influence on our analysis.

## Discussion

Using simulated sequence data and multiple regression analysis, we have shown that the prediction accuracy of recombination-detecting programs is affected by the reciprocal and non-reciprocal nature of the recombination event, prior evolutionary history, subsequent substitutions after the recombination event, and the choice of parameter settings in certain programs.

### Reciprocal versus non-reciprocal recombination events

The approaches showed higher accuracy in recovering reciprocal recombination events than non-reciprocal events, owing to the strength of the phylogenetic signal within the recombined region. In a reciprocal event, two lineages are exchanged, disrupting the phylogenetic relationship while retaining the original tree shape that has four terminal edges of equal length. In a non-reciprocal event, one sequence is over-written by another, reducing the genetic diversity in the set and producing a four-taxon tree in which two of the sequences are identical. This effect is stronger when all four sequences are more similar to each other at the point of recombination: while the region consisting two identical sequences was easily identified as a recombined region, a relatively small number of substitutions simulated afterward was sufficient to attenuate the phylogenetic signal of this region. Therefore, the phylogenetic signal of non-reciprocally recombined regions was more sensitive to subsequent substitutions than was that of the reciprocally recombined regions.

### Evolutionary history prior to recombination

The prior evolutionary history of the four sequences also affects the accuracy of a program in assigning the correct phylogeny to the recombined and non-recombined regions. Two good examples that illustrate this point are L50/05 and L05/50 in the non-reciprocal set (Figure 5).

In the case of L50/05, BARCE and DualBrothers showed high accuracy in recovering the phylogeny of the non-recombined region, but not of the recombined region. The phyletic grouping of sequences (1, 2) and sequences (3, 4) is supported by more shared substitutions in L50/05 than in L05/50, so the program can recover the non-recombined region more easily. The lower accuracy within the recombined region is due to the effect of topological structure as mentioned above when the sequences are more similar to each other, i.e. when *θ_2 _*is short. The exact opposite trend was observed in L05/50 (longer *θ_2_*), where the phylogenetic signal of the non-reciprocal event was stronger. Since *θ_1 _*was short in this case, the phyletic grouping of sequences (1, 2) and sequences (3, 4) is not as obvious as in the case of L50/05. Therefore, the programs showed low accuracy in recovering the non-recombined region, but high accuracy in recovering the recombined region. Similarly with the observation in L50 of the reciprocal set (Figure 4), recombination-detecting programs showed high accuracy in recovering the phylogeny of both the recombined and non-recombined regions when the recombining sequences are more divergent. This observation supports previous studies that suggested that recombination is easier to detect with increasing levels of sequence divergence [37,38], and that phylogenetic analyses of non-reciprocal recombination events are more sensitive to the prior evolutionary history of the sequences than are similar analyses of reciprocal events [28].

### Substitutions after the recombination event

The extent of substitution after the recombination event (*λ*) also plays an important role in determining the prediction accuracy of an approach. The phylogenetic signal of the recombined region becomes attenuated as subsequent evolution progresses, and the task of detecting evidence of ancient recombination events can be difficult or impossible. While all approaches in this study showed lower prediction accuracy with increasing amounts of subsequent substitution, the substitution distribution-based approach was found to be most sensitive. High rates of subsequent substitution independently on each sequence disrupt long contiguous matches in a local pairwise comparison, which the substitution distribution-based approach i.e. GENECONV is solely based on. By iteratively sampling phylogenies across the alignment and suppressing minor variations in the phylogenetic pattern across a set of sequences, the Bayesian phylogenetic-based approach was least sensitive to the effects of subsequent substitution. As RecPars is based on finding parsimonious topological changes across alignment columns, and GENECONV is based on significant pairwise alignments of fragments, these programs perform better when recombined regions are longer.

### Parameter settings

We demonstrated that the prediction accuracy of certain programs (e.g. RecPars) is highly sensitive to specific parameter settings. When dealing with real datasets, it is almost impossible to know in advance the optimal recombination cost to use in RecPars [38]. This supports the finding from a previous study that the phylogenetic approach performs poorly in detecting recombination [29]. A similar trend was observed in GENECONV, for which prediction accuracy was shown to be affected by the *gscale *setting, as described in the Results section.

### Identification of recombination breakpoints

Previous studies suggest that Reticulate and GENECONV have similar detection power in detecting recombination events [45], and that the compatibility approach of Reticulate is one of the more reliable methods [38]. Although these methods are computationally less intensive than the Bayesian phylogenetic-based approaches, some of them (e.g. Reticulate) were designed to detect the presence of recombination events, not to locate recombination breakpoints [29,37].

The Bayesian phylogenetic-based approach proved to be the best in delineating recombination breakpoints, showing high accuracy. This approach has a great advantage over the other approaches in detecting ancient recombination events, as the prediction accuracy was the least dependent on the extent of subsequent substitution. In many instances, BARCE showed a pattern of gradual transition from one topology to another, with intervening sites that have no dominant topology. This pattern is a good indicator of a recombination event, but the exact location of the breakpoint is not obvious. Although an abrupt change of tree topology BPP proposed between two adjacent sites is a good indication of a recombination breakpoint in some cases, there is no explicit proposal of breakpoints in BARCE. While the whole alignment was assumed to be under the same evolutionary pressure in BARCE, the dual Multiple Change-Point (MCP) model in DualBrothers [42], designed specifically to identify recombination breakpoints, proposes change point within the alignment, independently based on changes of tree topology and evolutionary rate. Although more false positives in defining phylogenies (e.g. L50/05 and L05/50 in Figure 5) were observed with DualBrothers, the explicit proposal of breakpoints in the program can be analysed in a more systematic manner. As shown in Figure 6, the breakpoints were identified with two sharp peaks in the graphs.

As is the case with other iterative MCMC approaches, the accuracy of BARCE comes at the expense of runtime, and the program can only be applied to datasets having exactly four sequences. DualBrothers can be applied to datasets with more than four sequences, but the program can be very time-consuming because the dual MCP model is described by eight parameters related to location of breakpoints, tree topologies and evolutionary rates.

### Combinatorial approach

Of all approaches examined, the conventional phylogenetic-based approach (RecPars) is of least practical use due to dependency of the program on parameter settings. Based on our findings in this study, we recommend use of the Bayesian phylogenetic-based approaches in detecting recombination events and breakpoints. Since the approach is time-consuming, faster approaches based on compatibility or substitution distribution can be used in the first instance to suggest the occurrence of a recombination event. For example, a marginally significant or significant clustering of incompatible sites in Reticulate or an unusually similar fragment (*e.g.*, with *p*-value ≤ 0.10) determined by GENECONV can be taken as suggesting a recombination event. Bayesian phylogenetic-based approaches, e.g. DualBrothers, can then be applied to confirm, in a more-accurate manner, the possible breakpoints in the recombination event. We assumed a homogeneous evolutionary regime throughout the course of sequence evolution in this study using simulated data. When the evolutionary scenario is more complicated e.g. with different rates and different evolutionary models within a tree [46,47], and for difficult detection problems involving highly divergent sequences, the use of fundamentally different approaches can provide multiple lines of evidence in support of the observed results.

## Conclusion

In this study, we highlighted the strengths and weaknesses of different classes of recombination detection programs. We demonstrated differences in phylogenetic signals within recombined and non-recombined regions, between a reciprocal and a non-reciprocal event, and how these signals affect prediction accuracy of different approaches in detecting occurrence and identifying breakpoints of a recombination event. Bayesian phylogenetic-based approaches showed high accuracy in identifying recombination breakpoints but are time-consuming due to the complexity of MCMC and the models used. The compatibility-based approach is fast and does not depend on specific parameter settings. The conventional phylogenetic-based approach, and to a lesser extent the substitution distribution-based approach, are sensitive to key parameter settings, and infer recombination events and breakpoints only when these settings are tuned to the data, which may be impossible to achieve with empirical data. In detecting recombination events, the negative dataset can be filtered out by a first-pass run using faster methods; the more-accurate (and slower) methods can then be used in delineating the recombination breakpoints among the positive dataset. The combinatorial approach is more time-efficient, especially when scanning through a large dataset. Since the methods applied here are different in principle, identification of an event by multiple methods may also increase our confidence that a recombination event has indeed occurred.

## Methods

### Simulation of sequence evolution

Seq-Gen [48] was used to generate four-taxon sequence sets of length 1000 nt using the HKY [49] model of substitution with nucleotide frequencies A = 0.20, C = 0.30, G = 0.30, T = 0.20, a transition/transversion ratio of 2, and a four-category discrete approximation to a gamma distribution of among-site rate variation with shape parameter alpha = 1.0.

The simulation process is illustrated in Figure 1. For simulating reciprocal recombination, the sequences were first evolved along the separate lineages, each with length *θ_1 _*of 0.05 or 0.50. For simulating non-reciprocal recombination events, sequences were first simulated using different combinations of *θ_1 _*and *θ_2 _*lineage lengths (0.05 and 0.50), in which *θ_1 _*and *θ_2 _*represent pre- and post-speciation lineage respectively. A reciprocal recombination event was simulated by manually exchanging a defined region between sequence 1 and sequence 3. A non-reciprocal recombination event was simulated manually by replacing a defined region of sequence 3 with that of sequence 1 as shown in Figure 1b. In all simulations, the recombined region was centred in the middle of the sequence block with 50% (500 nt) of the total sequence length (1000 nt), creating recombination breakpoints *r_1 _*(250/251) and *r_2 _*(750/751). After recombination, subsequent substitutions (*λ*) were simulated independently for each sequence with 0.00, 0.25 or 0.50 substitutions per site in each set. In all, 100 replicates were simulated for each test set.

### Detection of recombination events

GENECONV, a substitution distribution-based method, uses non-parametric statistics to rank possible recombination events in an alignment, in which pairwise polymorphic sites are compared and scored [36]. Recombination is inferred when sub-sequences in a two-sequence region are significantly more similar to each other than in the other regions in the sequence alignment. GENECONV version 1.81 was run with the simulated sequences in PHYLIP format as input. The parameter *gscale *= 1 was applied in all cases. Other default settings were used, *i.e. *minimum fragment length = 1, minimum number of polymorphisms = 2 and minimum pairwise score = 2.

RecPars is based on a parsimony algorithm that infers phylogenies for different segments in a sequence alignment; a recombination event can be inferred where these phylogenies change [40]. The assignment of incongruent topologies is affected by the recombination cost, which is the penalty associated with introducing a recombination breakpoint into the sequence. In this work, no recombined regions were detected when the recombination cost was set too high (*e.g. *when the default setting of 100 was used), while a great number of incongruent tree topologies were defined when little or no recombination cost (*e.g. *< 10) was applied. Therefore, for each analysis, recombination cost was initially set at *c *= 100, repeated with *c *– 1 and so forth, until the sequences were partitioned into three or more fragments. We took the maximum recombination cost that caused the sequences to be partitioned into three or more fragments to be the optimal recombination cost. RecPars was run with input sequences in RecPars format, with a uniform substitution cost of 1.

Reticulate is a compatibility-based method to detect phylogenetic incongruence within an alignment, based on parsimoniously informative sites [33]. The goal is to identify the tree(s) that provide the most parsimonious explanation (minimal number of substitutions) for each pair of sites, and then determine whether these trees are compatible with one another. Informative sites are defined as sites that have at least two different nucleotides present in two or more sequences each. Uninformative sites are discarded prior to analysis, since they cannot distinguish among tree topologies. The informative sites are paired and compared with each other. Two informative sites are considered compatible if both can be explained using the same phylogenetic tree. A matrix is generated in which each cell corresponds to the compatibility of pairs of informative sites. A cluster of incompatible sites in the matrix signifies a possible recombined region. Reticulate [33] was run with input sequences in FASTA format. We modified the source code to output the matrix directly in encapsulated postscript format, for the ease of large-scale batch runs. Clustering of sites was determined using neighbour similarity score (NSS) statistics incorporated in the program, with the generation of 1,000 random matrices for each simulation set.

BARCE, or Bayesian Application for Recombination and gene Conversion Estimation, is a program for detecting recombination breakpoints in alignments of four sequences [41]. Hidden Markov models are used to represent the patterns among different tree topologies assigned to each site (column) of the sequence alignment. Bayesian posterior probabilities are then assigned to all three possible tree topologies for each site. A proposed change of tree topologies between two adjacent sites suggests a recombination breakpoint. BARCE version 1.2 was run with sequences in PHYLIP format using the F84 [50] model, equally distributed prior probability of tree topologies at 1/3 each, difficulty of changing trees = 0.9, burn-in period = 100,000 generations, and length of sampling period = 100,000 generations. Initial character frequencies and transition/transversion ratios were estimated from the data.

DualBrothers is a Bayesian phylogenetic-based method for defining recombination breakpoints using a dual multiple change-point (MCP) model [42]. The spatial phylogenetic variation within the alignment is described by two independent change-point processes introduced by the dual MCP model based on changes in tree topology and evolutionary pressures across a set of sequences. The proposed changes are captured by a reversible-jump MCMC sampling algorithm [51] extended from a Metropolis-Hastings sampling scheme. DualBrothers samples parameters that define an HKY substitution model [49], and assumes that branch lengths are *a priori *independent across the tree and from site to site [52]. The algorithm can be applied to alignments with more than four sequences, but the sampling process is slow owing to the number of parameters considered.

A modified version of DualBrothers 1.1 was kindly provided by Aaron Darling (University of Wisconsin). The program was run with MCMC chain length = 550,000 generations, burnin = 50,000 generations, window_length = 5, Peter Green's constant [51] C = 0.20 and start_tree = (0,(1,(2,3))). Other parameters were run with default settings.

To assess the sensitivity of sampled breakpoint posterior probabilities to the choice of start point, we performed a replicated analysis of a subset of our simulated data sets. A set of sequences was chosen at random from each of the 18 combinations of event type (reciprocal or non-reciprocal event with combinations of *θ_1_*, *θ_2 _*and *λ*). The DualBrothers analysis as described above was performed ten times on each of these data sets, with each run commencing from a random starting point. The posterior probability of each site being proposed as a breakpoint was obtained for each replicate for comparison.

### Analysis of program accuracy

For GENECONV and RecPars, the average accuracy, determined separately for the recombined and non-recombined regions, was defined as the number of sites (columns) in the alignment that are correctly assigned to the expected topological relationship, divided by the total number of sites considered within the region:

average accuracy=(NRTR)+(NNRTNR)2
 MathType@MTEF@5@5@+=feaafiart1ev1aaatCvAUfKttLearuWrP9MDH5MBPbIqV92AaeXatLxBI9gBaebbnrfifHhDYfgasaacH8akY=wiFfYdH8Gipec8Eeeu0xXdbba9frFj0=OqFfea0dXdd9vqai=hGuQ8kuc9pgc9s8qqaq=dirpe0xb9q8qiLsFr0=vr0=vr0dc8meaabaqaciaacaGaaeqabaqabeGadaaakeaacqWGHbqycqWG2bGDcqWGLbqzcqWGYbGCcqWGHbqycqWGNbWzcqWGLbqzcqqGGaaicqWGHbqycqWGJbWycqWGJbWycqWG1bqDcqWGYbGCcqWGHbqycqWGJbWycqWG5bqEcqGH9aqpdaWcaaqaamaabmGabaWaaSaaaeaacqWGobGtdaWgaaWcbaGaemOuaifabeaaaOqaaiabdsfaunaaBaaaleaacqWGsbGuaeqaaaaaaOGaayjkaiaawMcaaiabgUcaRmaabmGabaWaaSaaaeaacqWGobGtdaWgaaWcbaGaemOta4KaemOuaifabeaaaOqaaiabdsfaunaaBaaaleaacqWGobGtcqWGsbGuaeqaaaaaaOGaayjkaiaawMcaaaqaaiabikdaYaaaaaa@5468@

in which *N*_*R *_is the number of sites that are correctly assigned within the recombined region, *N*_*NR *_is the number of sites that are correctly assigned within the non-recombined region, *T*_*R *_is the total number of sites within the recombined region and *T*_*NR *_is the total number of sites within the non-recombined region. This measure assigns equal weight to the recombined and non-recombined regions, regardless of their relative lengths. If the program correctly assigned a tree topology to all sites within a region but not to those sites within the other (for instance, when no recombination was detected), an average accuracy of 0.5 would be obtained. The average accuracy was scaled to a range of [-1,1] to yield the prediction accuracy, with 1.0 indicating perfect correlation between prediction and simulated history, and -1.0 indicating perfect anti-correlation:

prediction accuracy=2×(average accuracy−12)=(NRTR)+(NNRTNR)−1
 MathType@MTEF@5@5@+=feaafiart1ev1aaatCvAUfKttLearuWrP9MDH5MBPbIqV92AaeXatLxBI9gBaebbnrfifHhDYfgasaacH8akY=wiFfYdH8Gipec8Eeeu0xXdbba9frFj0=OqFfea0dXdd9vqai=hGuQ8kuc9pgc9s8qqaq=dirpe0xb9q8qiLsFr0=vr0=vr0dc8meaabaqaciaacaGaaeqabaqabeGadaaakeaacqWGWbaCcqWGYbGCcqWGLbqzcqWGKbazcqWGPbqAcqWGJbWycqWG0baDcqWGPbqAcqWGVbWBcqWGUbGBcqqGGaaicqWGHbqycqWGJbWycqWGJbWycqWG1bqDcqWGYbGCcqWGHbqycqWGJbWycqWG5bqEcqGH9aqpcqaIYaGmcqGHxdaTdaqadiqaaiabdggaHjabdAha2jabdwgaLjabdkhaYjabdggaHjabdEgaNjabdwgaLjabbccaGiabdggaHjabdogaJjabdogaJjabdwha1jabdkhaYjabdggaHjabdogaJjabdMha5jabgkHiTmaalaaabaGaeGymaedabaGaeGOmaidaaaGaayjkaiaawMcaaiabg2da9maabmGabaWaaSaaaeaacqWGobGtdaWgaaWcbaGaemOuaifabeaaaOqaaiabdsfaunaaBaaaleaacqWGsbGuaeqaaaaaaOGaayjkaiaawMcaaiabgUcaRmaabmGabaWaaSaaaeaacqWGobGtdaWgaaWcbaGaemOta4KaemOuaifabeaaaOqaaiabdsfaunaaBaaaleaacqWGobGtcqWGsbGuaeqaaaaaaOGaayjkaiaawMcaaiabgkHiTiabigdaXaaa@771D@

In RecPars, sites in an alignment that were assigned the expected topology were considered as correct assignments. In GENECONV, a full list of pairwise fragments was obtained for each simulated run, with a statistical significance assigned to each fragment based on the observed sequence similarity. Each site (column) within the original alignment was assigned the relationship implied by the pairwise fragment bearing the highest statistical significance. If the relationship assigned to an alignment site was consistent with the known topological relationship of the sequences, the site was recorded as having a correct assignment.

To examine the support for the topology of the recombined region in relation to the prediction accuracy of GENECONV and RecPars, the phylogeny of the recombined region in each sequence sets was determined by Bayesian inference using a Markov chain Monte Carlo (MCMC) approach implemented in MRBAYES [43], applying the K2P [53] model of substitution, and a discrete approximation to a gamma distribution with four categories. MCMC analysis was run for 1,100,000 generations, with burn-in = 100,000 generations, number of chains = 4 and temperature parameter for heating the chains = 0.5.

For Reticulate, clustering significance of pairwise comparison in the compatibility matrix *via *the neighbour similarity score (NSS) was used as the criterion of prediction accuracy. There are two possible colours in each cell, corresponding to the relationship between the alignment columns under consideration: incompatible (black) or compatible (white). The NSS ranged from 0.5 to 1.0, with higher NSS representing stronger clustering of similar tree topologies. Using a Monte Carlo approach in Reticulate, a total of 1000 random matrices was generated for each simulation set to test the non-randomness of clustering, *i.e. *its independence from the ordering of informative sites. If the clustering is non-random, the NSS values obtained from these random matrices should rarely be greater than the NSS obtained from the original matrix. The probability of a random matrix with an NSS greater than or equal to the NSS of the original matrix was interpreted as a *p*-value, in which clustering with *p *≤ 0.05 was considered non-random, and therefore significant.

Both BARCE and DualBrothers assigned each site (column) of the four-sequence alignment a BPP for possible tree topologies: (a,b),(c,d); (a,c),(b,d); or (a,d),(b,c). The predictive accuracy of BARCE was assessed at a series of BPP thresholds ranging from 0.5 to 1.0. For each threshold level, if any of the three topologies was given a BPP ≥ threshold level, the site was considered in the calculation of prediction accuracy; otherwise the prediction for that site was ignored. Prediction accuracy, determined separately for the recombined and non-recombined regions, was defined as the number of correct assignments over the total number of sites considered. If the expected tree topology was given the highest BPP at the particular site, the site was treated as a correct assignment. At higher threshold levels, the prediction accuracy is expected to be higher, as fewer sites are considered.

### Multiple linear regression analysis

Multiple linear regression (MLR) analysis was carried out using the statistical package R [54] to examine the significance of the relationship between the prediction accuracy of a program, and the simulation parameters that were varied in the analysis. The linear model used was:

Accuracy ~ Template + Brlen

Factors considered are: (i) starting tree topologies in non-reciprocal set or primary (ancestral) lineage length in reciprocal set (*Template*) and (ii) subsequent substitution after recombination event (*Brlen*). Analysis for GENECONV and RecPars also includes *Gscale *for gscale setting in GENECONV and *Recost *for recombination cost setting in RecPars. To test for violations of the assumptions in MLR analysis, the Kolmogorov-Smirnov test [55] for normality and Fligner-Killeen test [56] for homoscedasticity were carried out. Residuals versus fitted value and Cook's distance [57] plots were employed to test for linearity and influence of outliers.

## Authors' contributions

CXC designed and conducted the experiments, analysed the results and prepared the manuscript. RGB and MAR were involved in experimental design and supervised the study. All authors contributed to the final manuscript.

## References

[B1] Carpenter AT (1984). Meiotic roles of crossing-over and of gene conversion. Cold Spring Harb Sym Quant Biol.

[B2] Holliday R (1974). Molecular aspects of genetic exchange and gene conversion. Genetics.

[B3] Meselson MS, Radding CM (1975). General model for genetic recombination. P Natl Acad Sci USA.

[B4] Szostak JW, Orrweaver TL, Rothstein RJ, Stahl FW (1983). The double-strand-break repair model for recombination. Cell.

[B5] Sankoff D, Cedergren R, Abel Y (1990). Genomic divergence through gene rearrangement. Methods Enzymol.

[B6] Sankoff D (2003). Rearrangements and chromosomal evolution. Curr Opin Genet Dev.

[B7] Lawrence JG (1999). Gene transfer, speciation, and the evolution of bacterial genomes. Curr Opin Microbiol.

[B8] Milkman R (1997). Recombination and population structure in *Escherichia coli*. Genetics.

[B9] Papke RT, Koenig JE, Rodriguez-Valera F, Doolittle WF (2004). Frequent recombination in a saltern population of *Halorubrum*. Science.

[B10] Inagaki Y, Susko E, Roger AJ (2006). Recombination between elongation factor 1 alpha genes from distantly related archaeal lineages. P Natl Acad Sci USA.

[B11] Nielsen KM, Kasper J, Choi M, Bedford T, Kristiansen K, Wirth DF, Volkman SK, Lozovsky ER, Hartl DL (2003). Gene conversion as a source of nucleotide diversity in *Plasmodium falciparum*. Mol Biol Evol.

[B12] Striepen B, Pruijssers AJP, Huang JL, Li C, Gubbels MJ, Umejiego NN, Hedstrom L, Kissinger JC (2004). Gene transfer in the evolution of parasite nucleotide biosynthesis. P Natl Acad Sci USA.

[B13] Haubold B, Kroymann J, Ratzka A, Mitchell-Olds T, Wiehe T (2002). Recombination and gene conversion in a 170-kb genomic region of *Arabidopsis thaliana*. Genetics.

[B14] Bailey JA, Eichler EE (2006). Primate segmental duplications: crucibles of evolution, diversity and disease. Nat Rev Genet.

[B15] Aguileta G, Bielawski JP, Yang ZH (2004). Gene conversion and functional divergence in the beta-globin gene family. J Mol Evol.

[B16] Archibald JM, Roger AJ (2002). Gene duplication and gene conversion shape the evolution of archaeal chaperonins. J Mol Biol.

[B17] Kudla G, Helwak A, Lipinski L (2004). Gene conversion and GC-content evolution in mammalian Hsp70. Mol Biol Evol.

[B18] Miller HC, Lambert DM (2004). Gene duplication and gene conversion in class II MHC genes of New Zealand robins (Petroicidae). Immunogenetics.

[B19] Jeffreys AJ, May CA (2004). Intense and highly localized gene conversion activity in human meiotic crossover hot spots. Nat Genet.

[B20] Ochman H (2001). Lateral and oblique gene transfer. Curr Opin Genet Dev.

[B21] Thomas CM, Nielsen KM (2005). Mechanisms of, and barriers to, horizontal gene transfer between bacteria. Nat Rev Microbiol.

[B22] Beiko RG, Harlow TJ, Ragan MA (2005). Highways of gene sharing in prokaryotes. P Natl Acad Sci USA.

[B23] Gogarten JP, Townsend JP (2005). Horizontal gene transfer, genome innovation and evolution. Nat Rev Microbiol.

[B24] Posada D, Crandall KA, Holmes EC (2002). Recombination in evolutionary genomics. Annu Rev Genet.

[B25] Huson DH, Bryant D (2006). Application of phylogenetic networks in evolutionary studies. Mol Biol Evol.

[B26] Bryant D, Moulton V (2004). Neighbor-Net: an agglomerative method for the construction of phylogenetic networks. Mol Biol Evol.

[B27] Lawrence JG, Ochman H (1997). Amelioration of bacterial genomes: rates of change and exchange. J Mol Evol.

[B28] Posada D, Crandall KA (2002). The effect of recombination on the accuracy of phylogeny estimation. J Mol Evol.

[B29] Posada D, Crandall KA (2001). Evaluation of methods for detecting recombination from DNA sequences: computer simulations. P Natl Acad Sci USA.

[B30] Weiller GF (1998). Phylogenetic profiles: a graphical method for detecting genetic recombinations in homologous sequences. Mol Biol Evol.

[B31] Etherington GJ, Dicks J, Roberts IN (2005). Recombination Analysis Tool (RAT): a program for the high-throughput detection of recombination. Bioinformatics.

[B32] Hein J (1990). Reconstructing evolution of sequences subject to recombination using parsimony. Math Biosci.

[B33] Jakobsen IB, Easteal S (1996). A program for calculating and displaying compatibility matrices as an aid in determining reticulate evolution in molecular sequences. CABIOS.

[B34] Jakobsen IB, Wilson SR, Easteal S (1997). The partition matrix: exploring variable phylogenetic signals along nucleotide sequence alignments. Mol Biol Evol.

[B35] Bruen TC, Philippe H, Bryant D (2006). A simple and robust statistical test for detecting the presence of recombination. Genetics.

[B36] Sawyer S (1989). Statistical tests for detecting gene conversion. Mol Biol Evol.

[B37] Posada D (2002). Evaluation of methods for detecting recombination from DNA sequences: empirical data. Mol Biol Evol.

[B38] Wiuf C, Christensen T, Hein J (2001). A simulation study of the reliability of recombination detection methods. Mol Biol Evol.

[B39] Graham J, McNeney B, Seillier-Moiseiwitsch F (2005). Stepwise detection of recombination breakpoints in sequence alignments. Bioinformatics.

[B40] Hein J (1993). A heuristic method to reconstruct the history of sequences subject to recombination. J Mol Evol.

[B41] Husmeier D, McGuire G (2003). Detecting recombination in 4-taxa DNA sequence alignments with Bayesian hidden Markov models and Markov chain Monte Carlo. Mol Biol Evol.

[B42] Minin VN, Dorman KS, Fang F, Suchard MA (2005). Dual multiple change-point model leads to more accurate recombination detection. Bioinformatics.

[B43] Huelsenbeck JP, Ronquist F (2001). MRBAYES: Bayesian inference of phylogenetic trees. Bioinformatics.

[B44] Osborne JW, Waters E (2002). Four assumptions of multiple regression that researchers should always test. Pract Assess Res Eval.

[B45] Brown CJ, Garner EC, Dunker AK, Joyce P (2001). The power to detect recombination using the coalescent. Mol Biol Evol.

[B46] Spencer M, Susko E, Roger AJ (2005). Likelihood, parsimony, and heterogeneous evolution. Mol Biol Evol.

[B47] Lopez P, Casane D, Philippe H (2002). Heterotachy, an important process of protein evolution. Mol Biol Evol.

[B48] Rambaut A, Grassly NC (1997). Seq-Gen: an application for the Monte Carlo simulation of DNA sequence evolution along phylogenetic trees. CABIOS.

[B49] Hasegawa M, Kishino H, Yano TA (1985). Dating of the human ape splitting by a molecular clock of mitochondrial DNA. J Mol Evol.

[B50] Felsenstein J, Churchill GA (1996). A hidden Markov model approach to variation among sites in rate of evolution. Mol Biol Evol.

[B51] Green PJ (1995). Reversible jump Markov chain Monte Carlo computation and Bayesian model determination. Biometrika.

[B52] Suchard MA, Weiss RE, Dorman KS, Sinsheimer JS (2003). Inferring spatial phylogenetic variation along nucleotide sequences: a multiple change-point model. J Am Stat Assoc.

[B53] Kimura M (1980). A simple method for estimating evolutionary rates of base substitutions through comparative studies of nucleotide sequences. J Mol Evol.

[B54] The R project for statistical computing. http://www.r-project.org/.

[B55] Durbin J (1973). Distribution theory for tests based on the sample distribution function.

[B56] Conover WJ, Johnson ME, Johnson MM (1981). A comparative-study of tests for homogeneity of variances, with applications to the outer continental-shelf bidding data. Technometrics.

[B57] Cook RD (1977). Detection of influential observation in linear-regression. Technometrics.

